# Supplementary material for: Learning curve and surgical outcome of robotic assisted colorectal surgery with ERAS program
Source: Sci Rep. 2022 Nov 29;12:20566. doi: 10.1038/s41598-022-24665-w (PMC9709162; doi:10.1038/s41598-022-24665-w)
Supplement: Supplementary file 1 — Supplementary Information 1. [file 41598_2022_24665_MOESM1_ESM.zip › LSC Raw data-Ñ[▒K/086/O_ñw╝╨░O▒Kñσ.pdf]

正式摘要(電子簽章：000000195282621)

請選擇...reporter

臺中榮民總醫院

Operation Note

[redacted]

- 手術日期：2020/07/22
- 手術醫師：VS 林俊余 , CR 楊曙榮 , R 陳凱祥 , R 吳柏寬
- Anesthesia : GE
- Position : Lithotomy
- Aseptic agents : Soap , Betadine Alcohol , Betadine Aqua

Diagnosis

- Pre-operative diagnosis : s/p loop T with vulvulus.
- Post-operative diagnosis : Ditto with fecolis with mild T-colon ischemiac change

Surgical Indication

- removal of obstruction side

Operation

- laparoscopic left hemicolectomy

Finding

- 1. Much hard stool at T-colon distal to the ostomy with mild ischemiac change
  2. Some cloudy ascites at pelvics
  3. There was no visible liver metastasis, no visible peritoneal seeding
  4. Laparoscopic left hemicolectomy with side-to-side anastomosis, endo GIA 60/3.8x3, re-inforcement with silk: done.
  5. Vessel ligation: left branch of middle colic, left colic vessels and IMV.
  6. No drain tube

Procedure

- General endotracheal anesthesia was induced and then the patient was placed in lithotomy position. Preoperative antibiotics were given. A Foley catheter and nasogastric tube were placed. The abdomen and perineum was prepped and draped in standard sterile fashion. A small vertical incision was made near the umbilicus and a 12mm trochar was inserted into the peritoneal cavity by open method. Peumoperitoneum was established with CO2 inflation. Laparoscopy was inserted and then a 12mm trochars were inserted over RLQ of abdomen. Three 5mm trochars were inserted over RUQ, LUQ and LLQ of abdomen. Fecolis was noted at T-colon. After identification of IMV, the IMV was held by grasper and the peritoneum overlying the mesentery was then scored with electrocautery. Dissection was done above the Gerota's fascia and toward the splenic flexure. The left branch of middle colic artery and left colic artery were then identified, ligated with endoclips, and transected. Using electrocautery, omentum was freed from the transverse colon. The colon was then freed from its peritoneal attachments along the line of Toldt proximally from the splenic flexure and distally to the pelvic inlet. Splenic flexure and descending colon were mobilized. A small midline incision was made. The D-T colon was delivered into the incision and points of transection were selected proximally and distally. The bowel was divided and then the specimen was removed. The two ends of the bowel were checked and found to be viable, with excellent blood supply. The fat was gently cleared from the terminal 2-3 mm of the bowel ends. The two end of the bowel were brought into apposition and found to lie comfortably without excessive tension. Side-to-side anastomosis was performed with enod GIA 60/3.8mmx3. Interrupted 3-0 silk Lambert sutures were used to re-inforcement at outer layer. The anastomosis was checked and found to be intact and widely patent. The fascia of midline wound was closed with interrupted sutures of No.1 Vicryl. The fascia of the 12 mm trochar wound was closed with 1-0 Vicryl. The skin was close with 3-0 Nylon. The patient

- tolerated the procedure well and was brought to the post-anesthesia care unit in stable condition.
- Estimated blood loss: minimum
- 器械數量: 正確
- 紗布數量: 正確

Image

- PAINT
  - abdomen

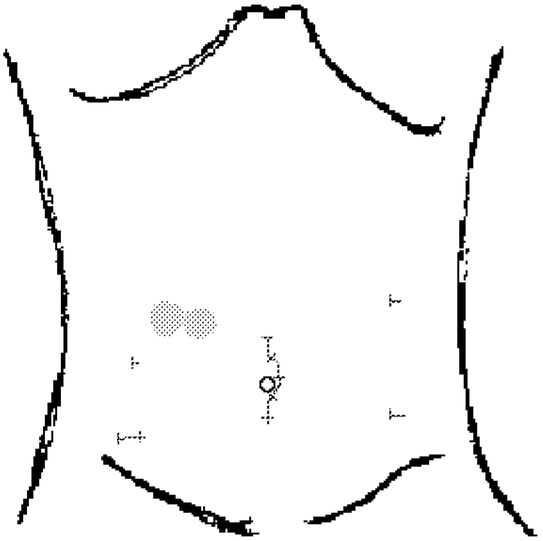

  - colon & rectum

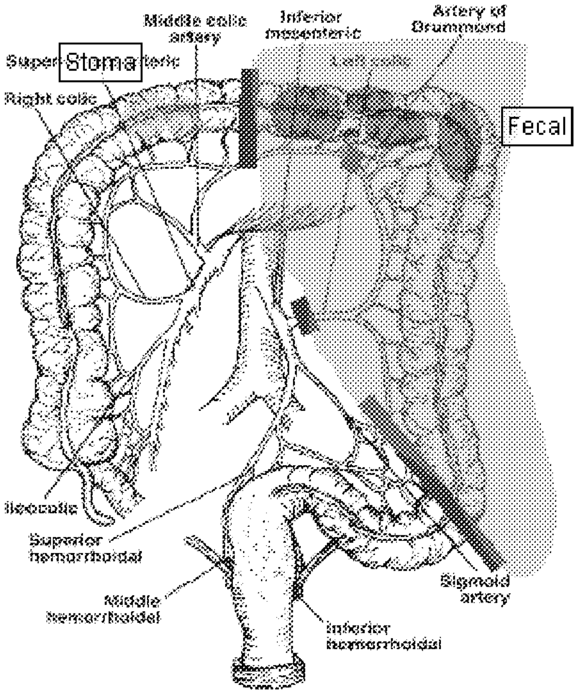

主治 2222H林俊余 / 醫師 2292G楊曙榮

正式摘要(電子簽章：**000000195282621)**

請選擇...reporter

doctorRank:B
